# A Comparison of the Plumage Condition of Three Egg-Laying Poultry Genotypes Housed in Non-Cage Systems

**DOI:** 10.3390/ani13020185

**Published:** 2023-01-04

**Authors:** Zofia Sokołowicz, Magdalena Dykiel, Jadwiga Topczewska, Józefa Krawczyk, Anna Augustyńska-Prejsnar

**Affiliations:** 1 Department of Animal Production and Poultry Products Evaluation, University of Rzeszów, Zelwerowicza Street 4, 35-601 Rzeszów, Poland; 2 Department of Food Production and Safety, Carpathion State College in Krosno, Rynek 1, 38-400 Krosno, Poland; 3 Department of Poultry Breeding, National Research Institute of Animal Production, Krakowska Street 1, 32-083 Balice Krakow, Poland

**Keywords:** alternative housing system, laying hen welfare, plumage status, native breed

## Abstract

**Simple Summary:**

The diverse types of cage-free systems require an understanding of the differences between them in terms of the welfare of the laying hens. One of the methods for assessing the welfare of laying hens is an assessment based on the plumage condition. In the conducted study, the plumage condition of laying hens raised in various types of alternative (non-cage) housing systems was assessed, i.e., in deep litter, free-range, and organic systems. The study included hens of the native Green-legged Partridges (Z-11), Rhode Island Red (R-11) hens covered by a genetic resource protection program, and hybrids of Hy-Line Brown at 20, 36, and 56 weeks of age. The type of cage-free system implemented had an effect on the condition of the plumage in the laying hens. Smaller losses of plumage were found in hens raised in free-range and organic farming conditions than in the litter system. As the age of the birds increased, the condition of the hens' plumage deteriorated. Rhode Island Red hens were characterised by the best plumage condition in litter housing conditions, while the native Green-legged Partridge hens showed the best plumage condition in the free-range and organic systems.

**Abstract:**

The study covered a total of 810 hens in 3 groups (housing systems) of 270 hens each. The plumage condition of laying hens raised in various types of alternative housing systems, i.e., in deep litter (B), free-range (FR), and organic systems (O), was assessed at 20, 36, and 56 weeks of age. The indoor stocking density was 6 hens/m^2^. The study included hens of the native Green-legged Partridge breed (Z-11), Rhode Island Red (R-11) hens covered by a genetic resource protection program, and hybrids of Hy-Line Brown. The plumage of the head, neck, back, tail, and abdomen was assessed on a 5-point scale. The assessment of individual hens' plumage was calculated as the sum of the scores of the head, neck, back, tail and abdomen and could range from 0 (no cover) to 20 points (full plumage). The type of alternative housing system implemented and the age of the laying hens had an effect on the plumage status of all body parts assessed (*p <* 0.05), while the genotype had an effect on the condition of the neck, back, and tail plumage (*p <* 0.05). In both the FR and O systems, the plumage status was similar and superior to that in B (*p <* 0.05). As the age of the birds increased, the condition of the hens' plumage deteriorated. The better state of the plumage in FR and O than in B may indicate improved levels of welfare in housing systems with access to outside runs.

## 1. Introduction

In accordance with the Council Directive 1999/74/EC [1], edible eggs in EU countries can currently be produced in cages (new type cages) and in deep litter, free-range, and organic systems. Consumer concern regarding the poor welfare of hens kept in conventional battery cages led to their ban in the EU (since 2012), the development of larger cages providing resources for key behaviours, and the development of alternative housing systems. However, a steady downward trend in the number of caged hens in favour of alternative systems has been observed in Europe for several years, and by 2030, a total ban is expected to be introduced on caged laying hens in all EU countries [2]. Improving welfare is important both for hens, egg producers, and consumers. Producers are often required to demonstrate that the welfare requirements for laying hens are being met. Consumers may motivate their purchase preferences with a concern for health, the environment, and animal welfare [3]. In response to the expectations of consumers interested in products obtained from animals raised under favourable conditions, producers are moving away from cage raising systems to alternative cage-free systems [4]. Alternative systems may be more hen-friendly mainly because they offer more space and opportunities for the hens to perform natural behaviours than cage systems [5,6,7,8]. However, the diversity of cage-free systems requires an understanding of the differences between the housing systems in terms of the welfare of laying hens, which, in turn, requires a reliable assessment of the welfare of the laying hens in the individual housing systems. The assessment of the welfare of hens should be non-invasive, comparable, and not costly [9]. The objective assessment and comparison of the welfare of birds raised in different types of alternative housing systems is a difficult task [10]. Among the many proposed methods of assessing the level of welfare of laying hens, it may be possible to estimate the welfare level on the basis of the results of the assessment of the condition of plumage [11]. A better condition of plumage indicates a higher level of well-being, and a poor condition suggests a low level. The plumage status of laying hens is also considered as an indicator of the health of the birds [12]. Deterioration of the condition of the plumage may have various causes, e.g., reflecting deficiencies in the composition of the feed [13] or injuries caused by equipment constituting the poultry house equipment [14]. The pecking of feathers is also considered an important cause of damage to plumage [15,16,17], which may indicate a reduced level of welfare of both the victims and perpetrators of pecking. Pecking is painful, and hens with already damaged feathers are more susceptible to further pecking and even cannibalism [18]. It was also found that pecking is associated with stress [19]. Pecking is a harmful behaviour which causes deterioration in the condition of the plumage and economic losses for egg producers. The loss of plumage also increases heat loss from the bodies of the birds [20]. In addition, hens with poor plumage condition increase their feed uptake to compensate for heat loss [21]. 

Different scoring systems are used to assess the condition of plumage [22]. The results of the assessment of the plumage of individual parts of the body or the combined result of the assessment of the condition of the plumage are used [23]. Usually, the results of the assessment of plumage of different parts of the body are summed up to obtain the result of the assessment of plumage of the whole body of an individual hen, or alternatively, the worst result of any part of the body is taken into account [24]. The average state of the plumage of the entire flock can be described by the average score [23] or the percentage of birds with particular results of the assessment of plumage [25]. The use of an average assessment (or sum) of whole-body plumage gives a good overall picture of the bird's plumage condition [26].

The prevalence of different types of alternative housing systems in contrast to cage systems raises the question of whether the welfare of laying hens in all types of alternative housing systems is the same. Therefore, the aim of the study was to assess the effect of the type of alternative housing system on the plumage condition of the Z-11 native breed of laying hens, R-11 hens covered by a genetic resources protection program, and Hy-Line Brown commercial hens.

## 2. Materials and Methods

### 2.1. Birds and Management

As the study did not involve any experimental manipulation or invasive procedures, it did not require the approval of an ethics committee. The study was conducted in Poland (EU) at the Experimental Station of the National Research Institute of Animal Production. The study covered a total of 810 hens, raised in 3 different alternative housing systems: deep litter (B group), free-range (FR group), and organic (O group). In total, 270 hens were allocated to each housing system, including 90 (3 subgroups of 30 pcs.) hens of the native breed of Green-legged Partridge hens (Z-11), 90 (3 subgroups of 30 pcs.) hens of the Rhode Island Red (R-11) breed covered by a genetic resources protection program in Poland, and 90 (3 subgroups of 30 pcs.) commercial Hy-Line Brown hybrids. Hy-Line Brown laying hens had their beaks trimmed at 10 days' age using the hot blade method. 

The breeding, hatching, and rearing up to the 16th week was carried out at the Experimental Station. The reared chicks were, in their 17th week, moved from their rearing rooms to the buildings where they were maintained throughout the entire period of use. Hens in each of the studied housing systems (B, FR, and O) were kept in a separate building belonging to the same experimental farm. Each henhouse was divided into compartments. B birds were kept in a henhouse with windows (window to floor ratio 1:15) on deep litter without access to an open-air run. Hens from the FR and O systems had free access to paddocks from 18 weeks of age. At 20 weeks of age, the average weight of R-11 hens was 1560 g, Z-11 1240 g, and Hy-Line Brown 1650 g. The population in the henhouse was 6 hens/m^2^. FR hens were kept in a henhouse with windows (ratio of window surface to floor surface 1:15) on deep litter with free access to grassy runs. The population in the henhouse was 6 hens/m^2^, while 4 m^2^ were allocated for each hen in the run. The hens of the O group were kept in accordance with the provisions on organic farming, i.e., Directive EC 1804/1999 [27] and Regulation (EEC) No 2092/91 of the Council of the European Economic Community [28]. The hens in this group were kept in a henhouse with windows (ratio of window surface to floor surface 1:15) on deep litter (6 hens/m^2^) with free access to a grassy run, with a density of 1 hen per 5 m^2^. In the FR and O henhouses, the hens had free access to the run through 40 × 45 cm openings located on the long wall of the building, from 6:00 to 20:00. The lighting program in the henhouse was the same for all groups and included 16 h of light and 8 h of darkness (16L:8D). During the period when the natural day was shorter than 16 h, daylight was supplemented with artificial light. In each of the assessed raising systems, the henhouses were equipped with round feeders, nipple drinkers, and nests. During the study period, the average temperature inside the henhouse was 15.1 ± 1.8 °C and on the outside run 13.5 ± 2.0 °C at 20 weeks of age, −2.4 ± 4.1 °C at 36 weeks of age, and 22.7 ± 1.9 °C at 56 weeks of age of the hens, respectively. 

Birds from the B and FR groups were fed ad libitum with a balanced diet for laying hens (16.08% protein, 11 MJ), and hens from the OS group were fed ad libitum with organic fodder for poultry (16.0% protein, 11 MJ).

### 2.2. Plumage Assessment

In total, 30 laying hens (3 subgroups × 10 hens) were randomly selected from each genotype (Z-11, R-11, and Hy-Line Brown) at 20, 36, and 56 weeks of age from each genotype for the evaluation of plumage status. Layers were 20 weeks old by autumn (September), 36 weeks old in winter (January), and 56 weeks old in early summer (June). At each time point, 270 laying hens (3 systems × 3 genotypes × 3 subgroups × 10 hens) were individually evaluated from each system. The state of the plumage was assessed for hens randomly picked from their individual compartments (subgroups). In order to avoid repeated assessments of the same birds, the assessed layers were placed in a separated part of the compartment for the duration of the assessment using an openwork or mobile partition, which was removed after the assessment of all birds was completed. The plumage condition of the five body parts was assessed on an ordinal 5-point scale developed and modified based on the LayWel project [12]. The following were assessed on a 5-point scale: plumage of the head, neck, back, tail, and abdomen, in which it was 4 points for complete/full plumage; 3 points for skin almost completely covered with feathers (bare patches ≤ 5 cm^2^); 2 points for incomplete cover, bare patches ≥ 5 cm^2^; 1 point for cover with significant losses but with several areas still covered (bare patches >5 cm^2^); and 0 points for total lack of cover. The assessment of the plumage of the whole body of a single hen was the sum of the ratings of the head, neck, back, tail, and abdomen and could range from 0 (no cover) to 20 (full plumage) points.

The assessment of the condition of plumage was carried out by 3 evaluators. All evaluators participating in the study were trained during previous pilot studies. The estimation of the assessor’s level of compliance (inter-rater reliability) was verified by Cohen's kappa coefficient. The compliance rates obtained were above 0.75.

### 2.3. Statistical Analysis

The obtained data were collated and subjected to statistical analysis using Statistica 13.3 [29]. The results on the influence of the breed, housing system, and age of laying hens on the assessment of the condition of plumage were verified using a non-parametric Kruskal–Wallis test. The share of hens with loss of plumage was expressed as a percentage. Differences were considered to be significant at *p <* 0.05.

Data on the effect, housing system, genotype, and age of laying hens on the plumage state were subjected to a multifactorial analysis of variance, and the main effects were determined (S—housing system effect, G—genotype effect, T—age effect) as well as the interaction between factors (S × G, G × T, S × T, S × G × T). The influence of the genotype, housing system, and age on the plumage status of hens was analysed using ANOVA, and in the analysis of the results, the following data were taken into account: the 3 systems (B, FR, O) × 3 genotypes (Green-legged Partridge hens (Z-11), Rhode Island Red (R-11), Hy-Line Brown) × 3 subgroups × 3 ages of the hens (20, 36, and 56 weeks of life) × 5 body parts (head, neck, back, tail, and abdomen) × 10 hens.

## 3. Results

The results of the scoring of the plumage condition (head, neck, back, tail, and abdomen) of the hens (Z-11, R-11, and Hy-Line Brown), aged 20, 36, and 56 weeks, raised in various types of alternative housing systems (B, FR, and O) are included in Table 1. The type of alternative housing system and the age of the laying hens had an effect on the plumage status of all body parts assessed (*p <* 0.05), while the genotype had an effect on the condition of the neck, back, and tail plumage (*p <* 0.05).

In our research, a significant effect of the housing system (*p <* 0.05) on the quality of the cover at 36 and 56 weeks of age was found, while the effect of the housing system on the state of the cover at 20 weeks of age of the laying hens was not confirmed (*p >* 0.05). The condition of the plumage in the free-range and organic systems at weeks 36 and 56 was similar and better than in the deep litter hens (Table 1 and Table 2).

In the initial period, i.e., at 20 weeks of age, in the B system, the plumage of the assessed breeds was full (4 points) or almost full (≥3.77). At the next assessment date, i.e., at 36 weeks of age, larger losses of plumage were recorded in all breeds observed, which to varying degrees covered all assessed areas of the plumage (Table 1). At the 56th week of life, the plumage condition of the laying hens of all assessed breeds deteriorated further. The losses concerned all the examined areas of the plumage and were the largest in the Z-11 hens on the head, back, tail, and abdomen (Table 1).

In the initial period in the FR system, i.e., in the 20th week of life, the plumage of the head and neck of the hens of the assessed breeds was full (4 points), while in the area of the tail and back, there were small losses in the Z-11 and R-11 hens, while in the Hy-Line Brown hens, plumage losses were also found on the abdomen. At the next assessment date, i.e., at 36 weeks of age in hens of all observed breeds, the loss of plumage was greater than at 20 weeks. The largest losses of plumage were found in the Hy-Line Brown hens, which covered all the examined areas of the plumage. At week 56, the plumage status of all breeds assessed improved compared to the plumage at week 36 (Table 1). 

In the initial period, i.e., in the 20th week of life in the O system, the plumage in hens of all assessed breeds was full or almost full, i.e., with small losses of feathers in the area of the tail (Z-11), or neck, back, and tail (R-11 and Hy-Line Brown). At the next assessment date, i.e., at 36 weeks of age in hens of all observed breeds, the loss of plumage was greater than at 20 weeks. The worst condition of the plumage at week 36 was found in the Hy-Line Brown hens and the best in the Z-11 hens. At week 56, the plumage status of all breeds observed improved compared to the plumage at week 36 (Table 1). 

The results of the assessment of the whole-body plumage of a single hen (the sum of points for the condition of the head, neck, back, tail, and abdomen) are presented in Table 2. It was found that the genotype had an effect on the plumage status of laying hens raised in the B and FR systems (*p <* 0.05), while in the O system no effect of the genotype was found on the plumage status of the laying hens (*p >* 0.05). The influence of the age of the laying hens on the condition of the feather cover in all types of housing was noted. 

**Figure 1:**
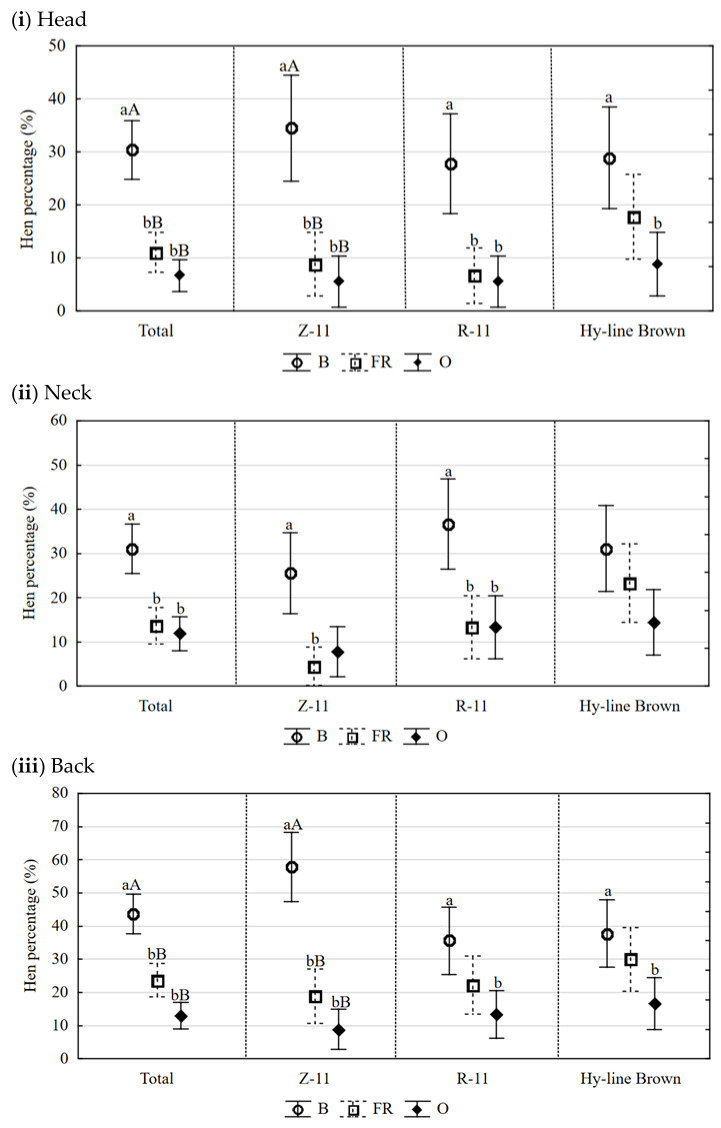

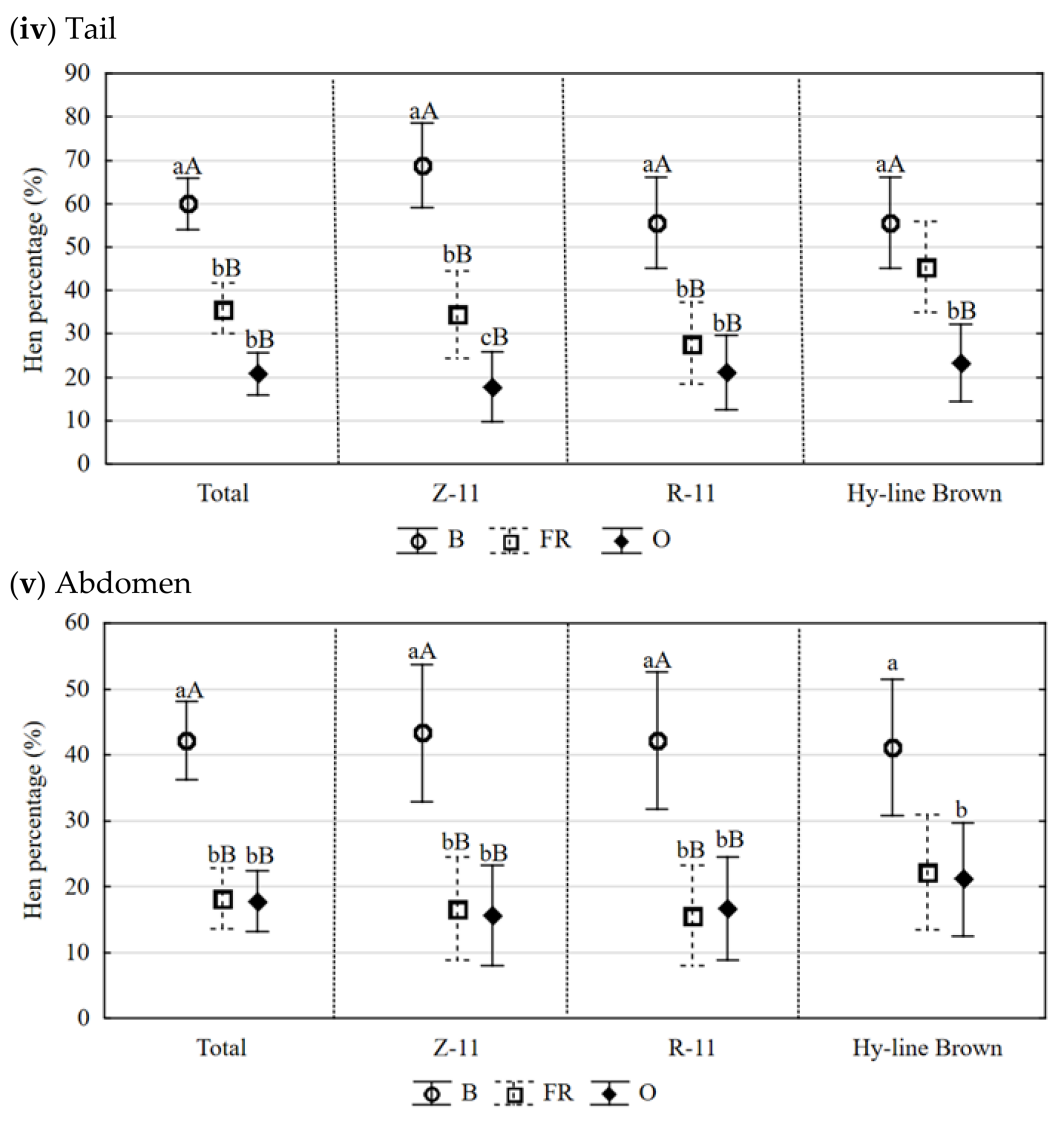
Effect of the type of alternative housing system on the share (%) of hens with feather defects in denoted parts of the body. Explanations: B—barn deep litter system; FR—free-range system; O—organic system; a, b, c—values in columns with various superscripts differ significantly within factors at *p <* 0.05; A, B —values in columns with various superscripts differ significantly within factors at *p <* 0.01. Total—for hens Z-11, R-11, and Hy-Line Brown.

The housing system had an effect on the share of hens with head, neck, back, abdomen, and tail defects in all assessed breeds (*p <* 0.01). The largest losses of plumage were observed in hens raised in the B system (Figure 1i–v).

## 4. Discussion

A key finding of this small research study was that birds with access to outdoor runs had better plumage than those confined indoors. Free range systems have the potential to be more animal-friendly, mainly because they offer the hens more space and opportunities to perform their natural behaviours than in barn systems. In the conducted research, the possibility of using the paddock probably translated into the hens’ better welfare, which corroborates the results of the studies by Bennett et al. [30] and Pettersson et al. [31]. 

Access to paddocks allowed the hens to move around and greatly increased their ability to engage in various behaviours, including active foraging and sunbathing, as also noted by Savory et al. [32]. Perhaps meeting the birds’ behavioural needs reduced their frustration and stress, thus preventing inappropriate behaviours such as pecking, as was also reported by Bestman and Wagenaar [23], Lambton et al. [33], and Sherwin et al. [34]. Similarly, results obtained by Shimmura et al. [35], Donaldson et al. [36], Dikmen et al. [37], and Szuman et al. [38] indicate that access to a paddock has positive effects on improving the plumage condition of laying hens. Szuman [38] reports that Green-legged Partridges in runs or restricted runs poor in greenery often present pterophagy and cannibalism and even the spontaneous loss of feathers, which may explain the worse plumage condition of the Z-11 layers in the B conditions than those of FR and O. The current study revealed that the feathering of the head and tail area in Hy-Line Brown hens in system B was worse than in system FR and O, which is consistent with the results obtained by Mahboub et al. [39] and De Haas et al. [40], who concluded that the risk of pecking in hens during the laying period is higher in the litter system than in the cage system. Perhaps the worse condition of the plumage in system B than in the systems FR and O can be explained by the lower frequency of aggressive behaviour in free range systems, as pointed out by Ferrante et al. [41]. In addition, in the studies by Sokołowicz et al. [42], the number of antagonistic behaviours in litter systems was higher than in the FR and O systems. Blatchford et al. [43] suggest that the most likely cause of loss of feathers in the aviary system may be pecking and aggression. These undesirable behaviours occur in all housing systems [44,45]. The pecking of flock members can be divided into gentle feather pecking, hard feather pecking, and cannibalism [46]. El-Lethey et al. [19] report that pecking is a misdirected behaviour related to feed intake, which develops in the absence of feeding opportunities and other important species needs. In our studies, the causes of the better plumage of hens in the FR and O systems can, therefore, be seen with access to a more diverse environment (the presence of grass on the run) by laying hens compared to the B system. Tahamtani et al. [47], Zepp et al. [48], and Geng et al. [49] have shown that diversification of the environment which enables the hens to engage in species behaviours can reduce the occurrence of aggression and pecking and thus improve the state of plumage both during rearing and laying. 

The differences in the condition of the plumage between the examined genotypes of hens found in our research probably result from the different tendencies of the laying hens of individual breeds to acts of aggression and pecking [50]. The influence of the genotype on antagonistic behaviours is reported by Sokołowicz et al. [42], Bolhuis et al. [51], and Uitdehaag et al. [52]. Additionally, in the studies of Campe et al. [53], the influence of the breed on the quality of plumage in aviary systems was found. According to Hocking et al. [54], pecking features related to the condition of the plumage are characterised by significant genetic variability. The main genes responsible for pecking were also found [55]. Contemporary commercial varieties of laying hens were selected mainly for high egg production and low feed use [10], with little regard to behavioural characteristics, as they were kept in cages in which the ability to express natural behaviours is limited [8]. It is believed that the selection of hens intended for commercial egg production led to an increase in the fearfulness of birds and a deterioration in their ability to cope with the new environment, affecting the degree to which they made use of the run [56,57]. The external environment exposes birds to potentially stressful situations, including weather conditions (rain, wind, extreme temperatures), parasites, diseases, and predation [58,59]. Studies have shown that hens kept with access to a run are less likely to peck feathers and have a better plumage condition [39,60,61,62]. However, the benefits that hens derive from access to the run largely depend on whether the birds choose to use it or not. Campbell et al. [63,64,65] and Larsen et al. [66] address the issue of frequency of use of the run and time spent there. Research by Sokołowicz et al. [42] showed that Z-11 hens used runs more often. Perhaps in the Z-11 hens that used the paddock more often, the pecking was directed at searching for and collecting feed and not at other individuals, which may explain the better condition of their plumage than the hens from commercial breeds. On the other hand, in Hy-Line Brown hens, the worse condition of the head and neck plumage may be associated with a higher frequency of aggressive behaviour, which in hens is most often directed at the head and neck. On the other hand, Hy-Line Brown hens had their beaks trimmed, which should have a positive effect on plumage condition as pecking problems are less common in hens with trimmed beaks, and their feather conditions are better than in hens with their beaks intact [33,67,68]. However, misbehaviours also occur in beak-trimmed flocks [69,70,71], so it can be assumed that beak-trimming did not completely prevent pecking problems in our study. Another factor that can be associated with differences in the plumage conditions of Z-11 hens and Hy-Line Brown hens may be the significant differences in the productivity of hens of the pure breeds Z-11 and R-11 and the hybrids of Hy-Line Brown. The high rate of egg-laying in commercial hybrids is the result of breeding selection in egg-laying breeding flocks in contrast to hens of native breeds which are, in keeping with the genetic resources conservation program, reared in small populations in which selection for greater egg production is not performed. Studies by Hagger et al. [72] indicate that hens with slight to moderate feather loss produced more eggs and higher egg mass than hens without any feather damage. The feed consumption of these better hens was higher, probably owing to their higher egg production.

In all observed alternative housing systems of hens in the initial laying period (in the 20th week), the laying hens were characterised by a good plumage condition, which, however, deteriorated at 36 weeks of age and with further deteriorated at 56 weeks in the laying hens raised on litter. As many authors point out [34,53,73,74,75,76,77,78], with the age of the laying hens, the state of plumage deteriorates in the following weeks of laying. At the same time, the deterioration of the plumage condition may be caused by pecking [45]. Campe et al. [53] showed a relationship between age and the state of plumage of hens during the period of use for both the entire body and for individual parts of the body, i.e., head and breast, while the remaining parts of the body, i.e., the neck, back, tail, and wings, were not dependent on age. The authors found that the older the birds were at the time of the assessment, the higher the percentage of hens with poor general plumage. Hinrichsen et al. [79] stated that the plumage of the back at the end of laying can be predicted on the basis of the plumage of the back and front parts of the bird during the peak period of laying. The reason for the deterioration of the plumage on the back and front parts of the bird (tail, abdomen, and neck) is different. Damage to the plumage on the back is usually associated with pecking [25,80], and the loss of feathers on the abdomen, which is also one of the first areas to be exposed [81], is also caused by their abrasion/wearing off [24,25]. Nicol et al. [44] noted the beneficial effect of increased open-air run utilization in reducing pecking. In our conducted research, the 56th week of life of the hens was in the spring period, in which the laying hens from the FR and O system used the runs more and more often and for longer. This can be considered as a probable reason for the improvement in the plumage of laying hens in the FR and O systems at week 56 compared to week 36, which occurred in the winter period when the use of the run by laying hens was lower [42]. The results of the research by Gen et al. [48] showed that greater use of the run is beneficial for the plumage and physical health of laying hens. Previous studies by Sokołowicz et al. [42] showed that Z-11 hens used the run to a greater extent than Hy-Line Brown hens, which may also explain the better condition of the plumage in Z-11 hens than Hy-Line Brown commercial hens. 

## 5. Conclusions

The better condition of the plumage in free-range and organic system conditions than in the deep litter system without access to a run may indicate a higher level of welfare in the free-range systems than in housing systems without access to an outside run. 

In both the free range and organic systems, hens of the Z-11 native breed and those of the R-11 breed covered by the genetic resources protection program were characterised by better plumage conditions than the commercial hens, which may indicate their better adaptation to free-range systems.

With age, the state of the hens' plumage deteriorated in all systems studied; however, in the free-range and organic systems, the pace of changes was lower than in the housing system on deep litter. The results obtained indicate that prevailing weather conditions or seasons may have indirect effects on the plumage, but this requires more extensive research.

## Figures and Tables

**Table 1:** The effect of the type of alternative housing system, genotype, and age of hens on the condition of plumage (on a scale of 0–4 points) of selected areas of the skin (0—very poor cover to 4—fully feathered).

Housing System ^1^	Genotype ^2^	Age of Laying Hens (wks) ^3^	Areas of the Skin				
			Head	Neck	Back	Tail	Abdomen
B	Z-11	20	4.00 ± 0.00 ^aA^	3.97 ± 0.18	3.90 ± 0.31 ^aA^	3.77 ± 0.43 ^aA^	3.93 ± 0.25 ^aA^
		36	3.37 ± 0.76 ^bB^	3.57 ± 0.63	2.97 ± 0.72 ^bB^	2.60 ± 1.00 ^bB^	3.10 ± 0.88 ^bB^
		56	3.23 ± 0.77 ^bB^	3.57 ± 0.63	2.63 ± 0.96 ^bB^	2.17 ± 1.05 ^bB^	3.10 ± 0.96 ^bB^
	R-11	20	3.97 ± 0.18 ^a^	3.97 ± 0.18 ^aA^	3.97 ± 0.18 ^aA^	3.83 ± 0.46 ^aA^	3.97 ± 0.18 ^aA^
		36	3.60 ± 0.56 ^ab^	3.33 ± 0.61 ^bB^	3.60 ± 0.50 ^abAB^	3.00 ± 0.83 ^bB^	3.57 ± 0.50 ^bA^
		56	3.40 ± 0.77 ^b^	3.53 ± 0.51 ^bB^	3.23 ± 0.68 ^bB^	2.90 ± 0.71 ^bB^	3.07 ± 0.58 ^cB^
	Hy-Line Brown	20	4.00 ± 0.00 ^aA^	4.00 ± 0.00 ^aA^	3.97 ± 0.18 ^aA^	3.80 ± 0.41 ^aA^	3.97 ± 0.18 ^aA^
		36	3.53 ± 0.68 ^bA^	3.57 ± 0.63 ^abAB^	3.33 ± 0.76 ^bB^	2.87 ± 0.97 ^bB^	3.30 ± 0.84 ^bB^
		56	3.37 ± 0.72 ^bB^	3.03 ± 1.00 ^bB^	3.33 ± 0.66 ^bB^	2.90 ± 0.92 ^bB^	3.07 ± 0.83 ^bB^
FR	Z-11	20	4.00 ± 0.00	4.00 ± 0.00	3.93 ± 0.25	3.73 ± 0.52 ^ab^	4.00 ± 0.00
		36	3.83 ± 0.67	3.90 ± 0.31	3.70 ± 0.47	3.33 ± 0.61 ^a^	3.63 ± 0.61
		56	3.90 ± 0.31	3.97 ± 0.18	3.80 ± 0.41	3.73 ± 0.58 ^b^	3.80 ± 0.41
	R-11	20	4.00 ± 0.00	4.00 ± 0.00	3.97 ± 0.18 ^a^	3.90 ± 0.40 ^a^	4.00 ± 0.00 ^a^
		36	3.87 ± 0.35	3.77 ± 0.50	3.50 ± 0.63 ^b^	3.47 ± 0.68 ^b^	3.63 ± 0.49 ^b^
		56	3.93 ± 0.25	3.73 ± 0.58	3.80 ± 0.41 ^ab^	3.67 ± 0.48 ^ab^	3.90 ± 0.31 ^ab^
	Hy-Line Brown	20	4.00 ± 0.00 ^a^	4.00 ± 0.0 ^a^	3.87 ± 0.35	3.67 ± 0.66	3.97 ± 0.18 ^a^
		36	3.63 ± 0.49 ^b^	3.60 ± 0.50 ^b^	3.47 ± 0.63	3.30 ± 0.65	3.33 ± 0.88 ^b^
		56	3.83 ± 0.38 ^ab^	3.70 ± 0.47 ^ab^	3.63 ± 0.50	3.40 ± 0.72	3.70 ± 0.60 ^ab^
O	Z-11	20	4.00 ± 0.00	4.00 ± 0.00	4.00 ± 0.00	3.90 ± 0.31	4.00 ± 0.00
		36	3.87 ± 0.35	3.87 ± 0.35	3.70 ± 0.65	3.67 ± 0.48	3.67 ± 0.61
		56	3.97 ± 0.18	3.90 ± 0.31	3.93 ± 0.25	3.87 ± 0.43	3.73 ± 0.58
	R-11	20	4.00 ± 0.00	3.93 ± 0.25	3.93 ± 0.25	3.93 ± 0.25	4.00 ± 0.00
		36	3.90 ± 0.31	3.80 ± 0.41	3.73 ± 0.45	3.60 ± 0.62	3.67 ± 0.55
		56	3.93 ± 0.25	3.87 ± 0.35	3.93 ± 0.25	3.67 ± 0.66	3.77 ± 0.50
	Hy-Line Brown	20	4.00 ± 0.00	3.93 ± 0.25	3.93 ± 0.25	3.90 ± 0.31	4.00 ± 0.00 ^a^
		36	3.83 ± 0.38	3.77 ± 0.50	3.67 ± 0.61	3.47 ± 0.82	3.53 ± 0.63 ^b^
		56	3.90 ± 0.31	3.83 ± 0.38	3.83 ± 0.38	3.63 ± 0.67	3.70 ± 0.53 ^ab^
*p*-value							
S ^1^			0.000	0.000	0.000	0.000	0.000
G ^2^			0.22	0.000	0.016	0.032	0.06
T ^3^			0.000	0.000	0.000	0.000	0.000
S ^1^ × G ^2^			0.21	0.78	0.000	0.000	0.35
G ^2^ × T ^3^			0.59	0.000	0.33	0.98	0.37
S ^1^ × T ^3^			0.000	0.020	0.000	0.000	0.000
S ^1^ × G ^2^ × T ^3^			0.84	0.27	0.027	0.08	0.53

Explanations: ^1^ housing system (S): B—barn deep litter system, FR—free-range system, O—organic system; S—effect of housing system; ^2^ G—effect of genotype; ^3^ T—effect of layer age; a, b, c—values in columns with various superscripts differ significantly within genotype at *p <* 0.05; A, B—values in columns with various superscripts differ significantly within genotype at *p <* 0.01.

**Table 2:** Total plumage (sum of head, neck, back, abdomen, and tail plumage) of laying hens from different types of alternative housing systems).

Housing System ^1^	Genotype ^2^	Age of Laying Hens (wks) ^3^			*p*-Value		
		20	36	56	G^2^	T^3^	G ^2^ × T ^3^
B	Z-11	19.57 ± 0.90 ^aA^	15.60 ± 2.90 ^bB^	14.70 ± 1.68 ^bB^	0.000	0.000	0.13
	R-11	19.70 ± 0.99 ^aA^	17.10 ± 2.32 ^bB^	16.13 ± 2.36 ^bB^			
	Hy-Line Brown	19.73 ± 0.64 ^aA^	16.20 ± 2.51 ^bB^	16.07 ± 2.60 ^bB^			
FR	Z-11	19.67 ± 0.61 ^aA^	18.40 ± 1.59 ^bB^	19.20 ± 1.47 ^abAB^	0.000	0.000	0.45
	R-11	19.87 ± 0.57 ^aA^	18.23 ± 1.85 ^bB^	19.03 ± 1.69 ^abAB^			
	Hy-Line Brown	19.50 ± 0.68 ^aA^	17.33 ± 1.75 ^bB^	18.27 ± 1.48 ^bB^			
O	Z-11	19.90 ± 0.31	18.77 ± 2.03	19.40 ± 1.38	0.25	0.000	0.93
	R-11	19.80 ± 0.66	18.70 ± 2.00	19.17 ± 1.62			
	Hy-Line Brown	19.77 ± 0.68 ^aA^	18.27 ± 1.95 ^bB^	18.93 ± 1.55 ^abAB^			
*p*-value							
S ^1^		0.24	0.000	0.000			
G ^2^		0.49	0.049	0.36			
S ^1^ × G ^2^		0.37	0.14	0.000			

Explanations: ^1^ housing system (S): B—barn deep litter system, FR—free-range system, O—organic system; S—effect of housing system; ^2^ G—effect of genotype; ^3^ T—effect of layer age; a, b—values in rows with various superscripts differ significantly at *p <* 0.05; A, B—values in rows with various superscripts differ significantly at *p <* 0.01.

## Data Availability

The data that support the findings of this study are available from the corresponding author (J.T.) upon reasonable request.
